# *De Novo* Germline Mutations in *SEMA5A* Associated With Infantile Spasms

**DOI:** 10.3389/fgene.2019.00605

**Published:** 2019-07-10

**Authors:** Qiongdan Wang, Zhenwei Liu, Zhongdong Lin, Ru Zhang, Yutian Lu, Weijue Su, Feng Li, Xi Xu, Mengyun Tu, Yongliang Lou, Junzhao Zhao, Xiaoqun Zheng

**Affiliations:** ^1^Department of Laboratory Medicine, The Second Affiliated Hospital and Yuying Children’s Hospital of Wenzhou Medical University, Wenzhou, China; ^2^School of Laboratory Medicine and Life Sciences, Wenzhou Medical University, Wenzhou, China; ^3^Institute of Genomic Medicine, Wenzhou Medical University, Wenzhou, China; ^4^Department of Pediatric Neurology, The Second Affiliated Hospital and Yuying Children’s Hospital, Wenzhou Medical University, Wenzhou, China; ^5^Department of Obstetrics and Gynecology, The Second Affiliated Hospital and Yuying Children’s Hospital, Wenzhou Medical University, Wenzhou, China; ^6^Key Laboratory of Laboratory Medicine, Ministry of Education, Wenzhou, Zhejiang, China

**Keywords:** epileptic encephalopathy, infantile spasms, *de novo* mutations, *SEMA5A*, whole-exome sequencing

## Abstract

Infantile spasm (IS) is an early-onset epileptic encephalopathy that usually presents with hypsarrhythmia on an electroencephalogram with developmental impairment or regression. In this study, whole-exome sequencing was performed to detect potential pathogenic *de novo* mutations, and finally we identified a novel damaging *de novo* mutation in *SEMA5A* and a compound heterozygous mutation in *CLTCL1* in three sporadic trios with IS. The expression profiling of *SEMA5A* in the human brain showed that it was mainly highly expressed in the cerebral cortex, during the early brain development stage (8 to 9 post-conception weeks and 0 to 5 months after birth). In addition, we identified a close protein-protein interaction network between *SEMA5A* and candidate genes associated with epilepsy, autism spectrum disorder (ASD) or intellectual disability. Gene enrichment and function analysis demonstrated that genes interacting with *SEMA5A* were significantly enriched in several brain regions across early fetal development, including the cortex, cerebellum, striatum and thalamus (q < 0.05), and were involved in axonal, neuronal and synapse-associated processes. Furthermore, *SEMA5A* and its interacting genes were associated with ASD, epilepsy syndrome and developmental disorders of mental health. Our results provide insightful information indicating that *SEMA5A* may contribute to the development of the brain and is associated with IS. However, further genetic studies are still needed to evaluate the role of *SEMA5A* in IS to definitively establish the role of *SEMA5A* in this disorder.

## Introduction

Epileptic encephalopathies (EEs) are a group of complex brain disorders characterized by intractable and early-onset epilepsy with or without developmental delays, which are highly genetically heterogeneous (Berg et al., 2010; Scheffer et al., 2017). Infantile spasms (IS, also known as West syndrome) are considered a subset of EEs that are characterized by the early onset of epileptic spasms, typically in the first year of life, and are always accompanied by a hypsarrhythmia pattern on the electroencephalogram (EEG) and developmental impairment (Nabbout and Dulac, 2003; Pavone et al., 2014). Moreover, EEs are caused by a variety of etiologies that are not yet fully understood. However, recent studies have provided evidence that genetic factors have a tremendous impact on the pathogenesis of EEs (McTague et al., 2016; Shbarou and Mikati, 2016). The discovery of candidate genes will further our understanding of the mechanisms underlying epileptogenesis.

The development of next-generation sequencing techniques, such as whole-exome sequencing (WES), has greatly facilitated gene discovery in EEs, and these approaches have been widely used to detect pathogenic mutations (Dimassi et al., 2016; Jin et al., 2018). Furthermore, recent genetic studies have used trio exome sequencing to confirm that many pathogenetic* de novo* mutations (DNMs) are critical genetic components in the pathogenesis underlying EEs (Claes et al., 2001; Carvill et al., 2013; Epi4K Consortium and Epilepsy Phenome/Genome Project, 2013). DNMs are the most damaging form of rare genetic mutations and occur mainly in the germline (Veltman and Brunner, 2012). Most genes with pathogenetic DNMs in EE encode voltage-gated ion channels or receptors associated with neurotransmitter, including sodium channels, potassium channels, GABA receptors, glutamate receptors and NMDA receptors (Fukata and Fukata, 2017; He et al., 2019), and are often involved in a variety of functional pathways related to neuronal excitability or synaptic and neuronal connectivity (Pardo et al., 2014).

Axon guidance proteins, including semaphorins, ephrins, slits, repulsive guidance molecules, and netrins, can act as attractants or repellents during axon branching, synapse formation and plasticity (Shen and Cowan, 2010; Van Battum et al., 2015). In the central nervous system, semaphorins play a role in synaptic plasticity including the regulation of synaptic structures and synaptic transmission (Pasterkamp and Giger, 2009). Moreover, both NMDA and AMPA receptors participate in synaptic plasticity (Song and Huganir, 2002; Lau and Zukin, 2007). Strikingly, DNMs have been identified in the *GRIN1* and *GRIN2B* genes, which encode the proteins that form the subunits of the NMDA receptor, in individuals with West syndrome and a severe developmental delay (Epi4K Consortium and Epilepsy Phenome/Genome Project, 2013; Lemke et al., 2014). Because appropriate brain function and mental health rely on the accurate regulation of the central nervous system synapse density, an imbalance between excitatory and inhibitory synaptic transmission is partly responsible for neurodevelopmental disorders such as schizophrenia and autism spectrum disorder (ASD) characterized by impairments in social interactions and restricted behaviors and interests (Penzes et al., 2011).

In this study, we performed WES on three individuals with IS as well as their unaffected parents and identified a novel damaging DNM in *SEMA5A*. As a member of the semaphorin gene family with bifunctional axon guidance activities, the discovery of a mutation in *SEMA5A* may further strengthen the role of axon guidance proteins in EEs. In addition, we also detected a DNM in *PLEKHG4B* and a compound heterozygous mutation in *CLTCL1* in patients with IS.

## Materials and Methods

### Subjects

Three probands with IS and their healthy parents were enrolled in the study from the Second Affiliated Hospital and Yuying Children’s Hospital of Wenzhou Medical University, after study approval was provided by the Hospital Ethics Committee. Moreover, written informed consent of all participants was obtained from their parents or guardians at the time of recruitment. All probands were referred with typical seizure presentation and were diagnosed with IS by an experienced pediatric neurologist.

### Whole-Exome Sequencing

Peripheral blood (2 ml) was drawn from the three affected probands and their unaffected family members. Genomic DNA was isolated using a QIAGEN DNeasy Blood & Tissue Kit (Qiagen, Valencia, CA, USA) from the peripheral blood of each included individual. The DNA was then subjected to an additional quality and quantity evaluation step using a NanoDrop 2000 spectrophotometer (Thermo Scientific, Wilmington, DE, USA). Subsequently, exome-coding DNA was captured with an Agilent SureSelect Human All Exon v6 Kit (Agilent Technologies, Santa Clara, CA, USA), and the libraries were sequenced on an Illumina HiSeq2000 sequencer (San Diego, CA, USA), which produced 150-bp paired-end reads.

All raw sequencing data obtained from these three trios were analyzed in a similar manner according to a customized bioinformatics pipeline (Wang et al., 2013). After quality filtering was performed on the raw sequence data using the Trim Galore program, the cleaned sequences were aligned to the human reference genome (GRCH37/hg19) using Burrows-Wheeler Aligner (BWA). Picard was performed to realign the reads and remove any reads that were duplicated or mapped to multiple genome locations. Variant and genotype calling were performed using the Genome Analysis Toolkit (GATK) (McKenna et al., 2010), and DNMs were detected by two software tools [ForestDNM (Michaelson et al., 2012) and mirTrios (Li et al., 2015)].

### Mutation Annotation

ANNOVAR was used to annotate all called variants. The minor allele frequency (MAF) of the detected sequence variants was estimated in various publicly available databases, including ExAC, UK10K, dbSNP147, 1000 Genomes, and ESP6500. If a detected variant was presented with a MAF > 0.1% in any database, it was eliminated. Subsequently, the effects of the detected variants were predicted according to SIFT (https://sift.bii.a-star.edu.sg/, a variant with SIFT score < 0.05 predicted damaging), VEST3 (https://karchinlab.org/apps/appVest.html, a variant with VEST3 score ≥ 0.5 indicated damaging), Polyphen2 (http://genetics.bwh.harvard.edu/pph2/, a variant with a score of 0.909 to 1.0 indicated probable damage, while those with scores between 0.0 to 0.446 meant benign), and GERP++ (http://mendel.stanford.edu/SidowLab/downloads/gerp/, a variant with a GERP++ score ≥ 2 indicated conserved). All potential damaging DNMs were visualized by Splicing Viewer (Liu et al., 2012) and confirmed by Sanger sequencing.

### Gene Expression

The RNA sequencing data for human tissue-derived *SEMA5A* were obtained from the human protein atlas (HPA) (https://www.proteinatlas.org). In addition, data were obtained from the human brain transcriptome at HBT (http://hbatlas.org/) to evaluate the spatial and temporal expression pattern of *SEMA5A* in human brain tissues. Furthermore, a digital atlas of gene expression patterns in mouse tissues at embryonic day 14.5 was selected and assessed in GenePaiant (www.genepaint.org). Expression patterns were defined by non-radioactive *in situ* hybridization (ISH), and selected images were annotated in detail.

### Construction of the Protein–Protein Interaction Network and Enrichment Analysis of Genes in the Network

A critical assessment and integration of protein–protein interaction (PPI) information was obtained from the STRING database to create the PPI networks. Then, we selected genes that interacted with *SEMA5A* and that had interaction scores greater than 200. Specific expression analysis (SEA) was performed on a developed online tool (http://genetics.wustl.edu/jdlab/csea-tool-2/) to explore whether *SEMA5A* and the identified interacting genes were highly enriched in a particular human brain region. In addition, R package clusterProfiler and DOSE were used to further explore the functions of the interacting genes in the PPI network. There were 43 *SEMA5A*-interacting genes that were shared by neuropsychiatric disorders and used to construct the interconnected PPI network. Random simulations of 100,000 permutations for genes and connections were performed to verify the non-random nature of the networks. Moreover, fisher’s exact test was used to evaluate the enrichment of 43 genes in the postsynaptic density (PSD) proteins (Collins et al., 2006) and genes under evolutionary constraint (Samocha et al., 2014).

## Results

### Identification of Potential Mutations

To detect candidate pathogenic DNMs, we recruited three trios with IS for WES. After low-quality reads and adapters were removed, approximately 5.2–11.03 GB of clean reads were obtained for each individual. All samples passed the quality control measures, and more than 99% of the qualified bases were aligned to the human reference genome (hg19) with an average sequencing depth of 74.91-fold. It is important to note that a mean of 87.49% of the target regions was covered at 20× and that more than 90% of the exonic regions were sequenced at 10× coverage (**Supplementary Table S1**). Following alignment and variant annotation, we detected a total of five *de novo* SNVs and three *de novo* indel mutations in coding regions. We confirmed that two DNMs and a compound heterozygous mutation were present among the three trios. In addition, all of the identified mutations were confirmed by Sanger sequencing, and the deleteriousness of mutations were predicted by SIFT, VEST3, Polyphen2, and GERP++.

### Candidate Genes for Infantile Spasms

**Figure 1:**
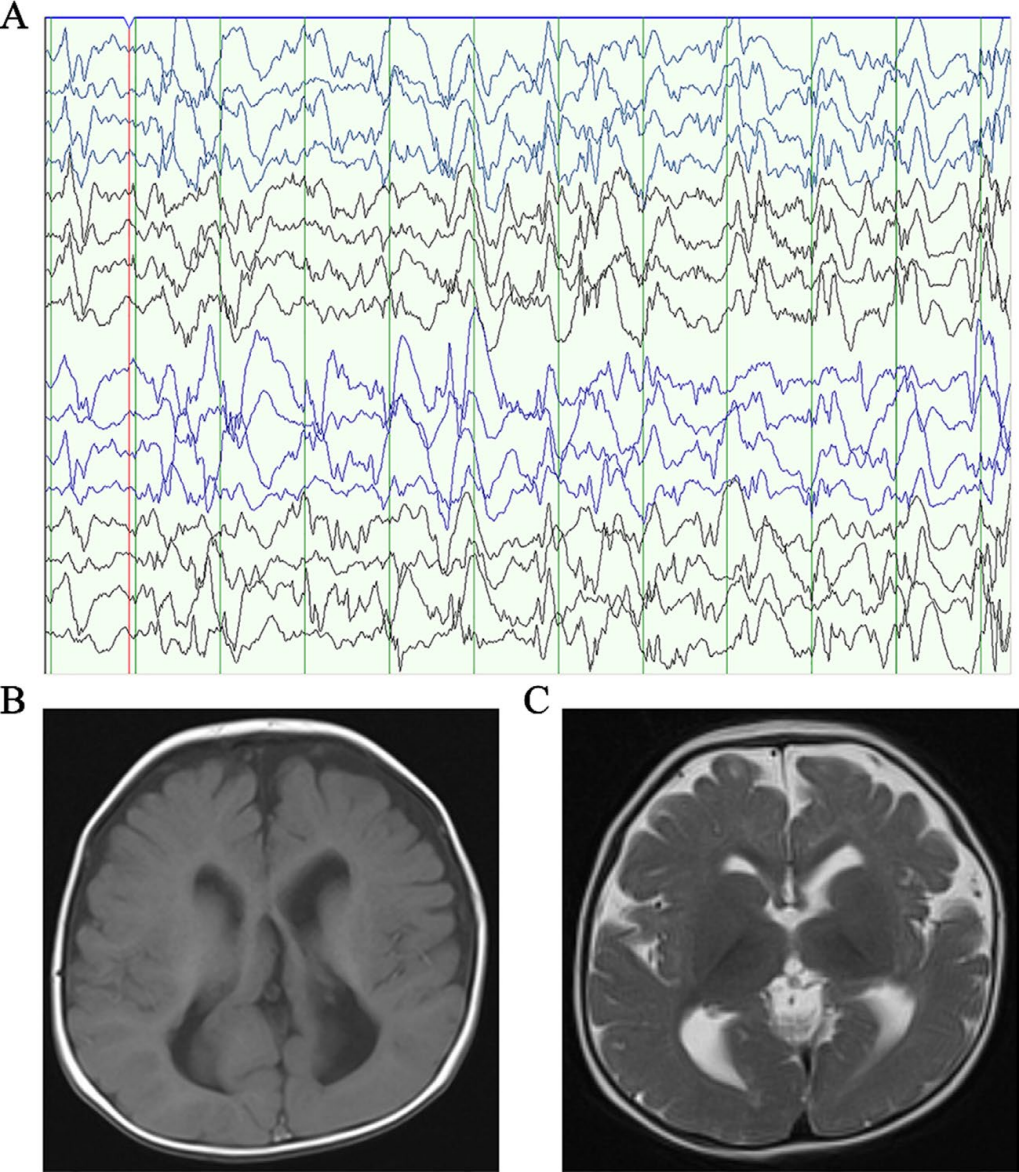
Clinical presentation. **(A)** The electroencephalogram obtained in patient 1 when he was 7 months old. **(B)** Axial T1-weighted brain MRI obtained in patient 1 when he was 7 months old. **(C)** Axial T2-weighted brain MRI obtained in patient 1 when he was 7 months old.

**Figure 2:**
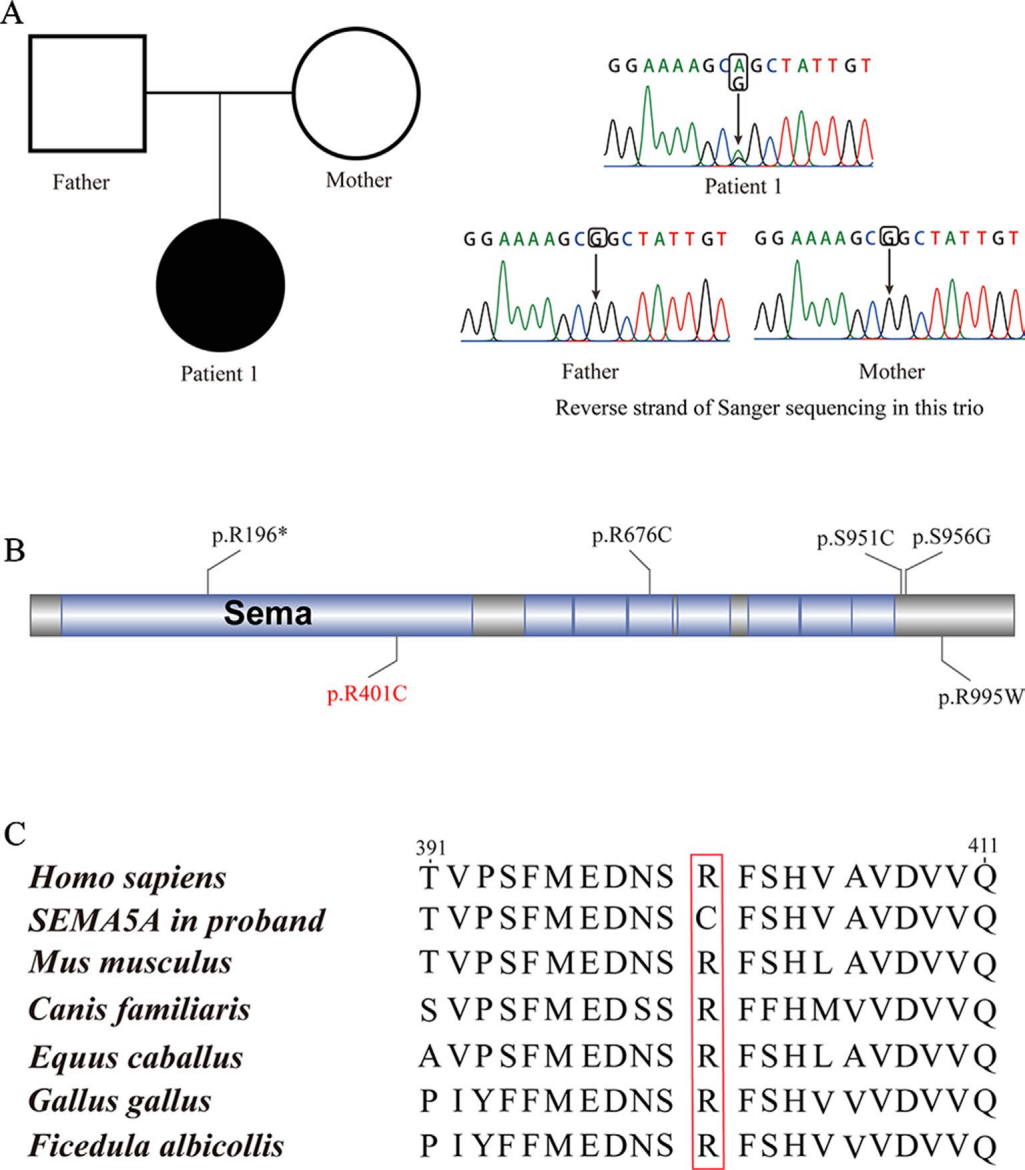
*De novo* mutations (DNMs) in *SEMA5A*. **(A)** Sanger sequencing of patient 1. The filled symbol indicates the affected individual. **(B)** Protein schematic of SEMA5A. Red indicates the DNM identified in this study, and black indicates mutations detected in ASD. **(C)** The conservation of the DNM in *SEMA5A* among various vertebrates.

Patient 1, who was born at term following an uneventful pregnancy to healthy non-consanguineous parents with no pertinent family history, had seizure symptom onset at the age of 7 months with the EEG displaying hypsarrhythmia (**Figure 1A**), a characteristic feature of IS. Additionally, the magnetic resonance imaging (MRI) of patient 1 at 7 months old, revealed brain dysplasia and agenesis of the corpus callosum (**Figures 1B, C**). The patient carried one missense mutation (c.1201C > T) in the *SEMA5A* gene that was identified by WES and confirmed as a DNM by Sanger sequencing (**Figure 2A**). At the structural level, the *de novo* missense mutation caused an arginine to cysteine substitution at amino acid 401 in the Sema domain [p.(Arg401Cys)], a conserved domain containing 450 amino acids (**Figure 2B**). The Sema domain serves as a repulsive guidance cue and might prevent axons from branching into surrounding myotome regions (Hilario et al., 2009). Then we predicted the structural model of the SEMA5A and there was no hydrogen bond between Arg-401 of SEMA5A and other residues. However, Cys-401 forms three hydrogen bonds with Phe-402, Asp-425, and Gln-292, respectively (**Supplementary Figure S1**). Hence, the substitution of arginine by cystine at amino acid 401 of SEMA5A may affect the structural stability.

In addition, we found that the identified DNM is evolutionarily conserved across various vertebrates (**Figure 2C**). Moreover, in silico prediction programs (SIFT, VEST3, Polyphen2 and GERP++) indicated that the *de novo* missense mutation in *SEMA5A* identified in this study is likely to be damaging and conserved in the protein (**Table 1**). Furthermore, the missense Z score of *SEMA5A* is 1.6794, which means that it tended to be intolerant of functional genetic variation (Lek et al., 2016). It should be mentioned that five different mutations, including a *de novo* missense mutation [c.2852C > G, p.(Ser951Cys)], a nonsense mutation [c.586C > T p.(Arg196*)] and three missense mutations, have been found in *SEMA5A* in individuals diagnosed with ASD (**Figure 2B**) (Iossifov et al., 2014; D’Gama et al., 2015; Mosca-Boidron et al., 2016). Additionally, *SEMA5A* has not been reported to be involved in epilepsy and is absent in any gene database related to epilepsy (Ran et al., 2015).

**Table 1:** Mutations detected by WES in this study in patients with IS.

Trio	Chr	Gene	Mutation	Inheritance	Protein change	SIFT (score)	VEST3 (score)	Polyphen2 (score)	GERP++ (score)
Patient1	chr5	*SEMA5A*	missense	*De novo*	p.(Arg401Cys)	Damaging (0)	Damaging (0.944)	Probably damaging (0.998)	Conserved (5.75)
Patient 2	chr22	*CLTCL1*	missense	inherited	p.(Met1316Val)	Tolerable (0.14)	Damaging (0.634)	Probably damaging (0.994)	Conserved (3.5)
Patient2	chr22	*CLTCL1*	missense	inherited	p.(Arg1165Cys)	Damaging (0)	Damaging (0.765)	Probably damaging (0.961)	Conserved (3.16)
Patient3	chr5	*PLEKHG4B*	missense	*De novo*	p.(Ala914Thr)	Tolerable (0.51)	Tolerable (0.118)	Benign (0.364)	Non-conserved (0.11)

We additionally identified a compound heterozygous mutation in *CLTCL1* in patient 2 (**Supplementary Figure S2A**). In this patient, one missense mutation was inherited from the mother [c.3946A > G, p.(Met1316Val)], and was predicted to be damaging and conserved by VEST3, Polyphen2 and GERP++. Another missense mutation [c.3493C > T, p.(Arg1165Cys)] that was inherited from the father was predicted by four prediction tools (SIFT, VEST3, Polyphen2, and GERP++) to be damaging and conserved (**Supplementary Figure S2B**, **Table 1**). Both mutations were located in a highly conserved domain (**Supplementary Figure S2C**).

In patient 3, we identified a missense mutation in *PLEKHG4B* [c.2740G > A, p.(Ala914Thr)] that was regarded as a DNM and confirmed by Sanger sequencing. Nevertheless, the SIFT, VEST3 and Polyphen2 tools predicted that this mutation is tolerable and benign, and GERP++ predicted that it is non-conserved. Therefore, it was not considered as a possible candidate mutation in further analyses.

### The Expression Profiles of SEMA5A

**Figure 3:**
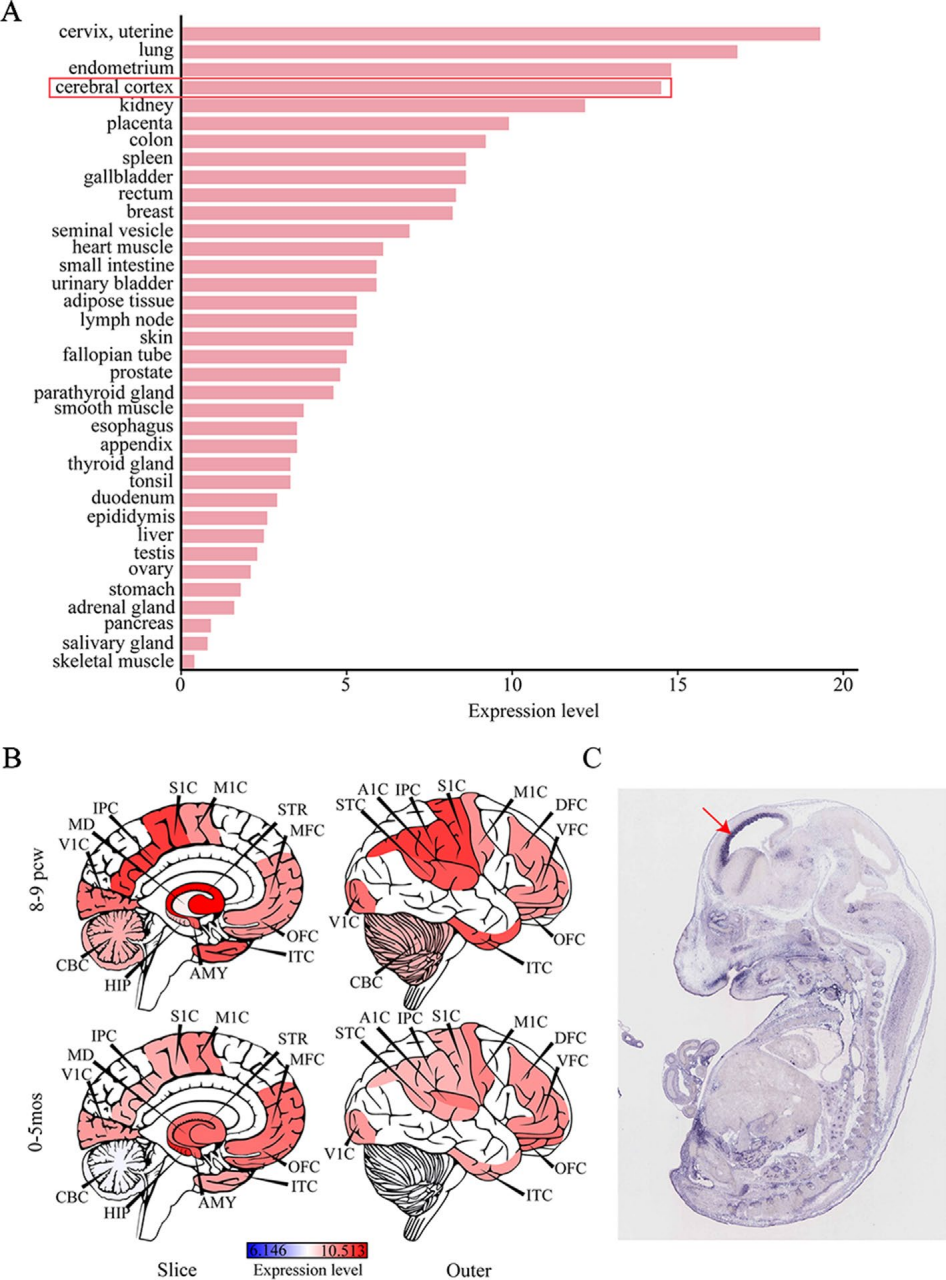
The expression profile of *SEMA5A*. **(A)** The expression of *SEMA5A* in 36 tissues in the human protein atlas (HPA) database. **(B)** Spatiotemporal expression profile of *SEMA5A* in HBT. A1C, primary auditory cortex; AMY, amygdala; CBC, cerebellar cortex; DFC, dorsolateral prefrontal cortex; HIP, hippocampus; IPC, posterior inferior parietal cortex; ITC, inferior temporal cortex; M1C, primary motor cortex; MD, mediodorsal nucleus of the thalamus; MFC, medial prefrontal cortex; OFC, orbital prefrontal cortex; S1C, primary somatosensory cortex; STC, posterior superior temporal cortex; STR, striatum; V1C, primary visual cortex; VFC, ventrolateral prefrontal cortex. **(C)** The expression of *SEMA5A* in a mouse embryo at embryonic day 14.5 was analyzed by situ hybridization. The arrow indicates the neocortex.

Considering that IS can give rise to severe cognitive and behavioral impairments, we attempted to determine the role of *SEMA5A* in the development of brain tissues by evaluating its expression pattern. RNA sequencing data were available for 36 tissues in the HPA database and indicated that *SEMA5A* acts in a variety of tissues. It is important to note that *SEMA5A* is highly expressed in the cerebral cortex, in which it showed its fourth-highest expression level in the 36 tissues (**Figure 3A**). To affirm this finding and to gain a higher-resolution spatiotemporal view of *SEMA5A* expression in the human brain, we analyzed RNA-seq data across various brain regions and developmental stages in HBT. The results showed that *SEMA5A* expression is extensively scattered across different developmental periods and regions of the human brain. Interestingly, *SEMA5A* is preferentially highly expressed in 11 areas of the neocortex, as well as several other brain regions such as the hippocampus (HIP), amygdala (AMY), and the striatum (STR), during early embryonic development (8 to 9 post-conception weeks, **Figure 3B**). In addition, *SEMA5A* was also highly expressed in almost all human brain tissues at 0 to 5 months after birth, which also represents an important stage of human brain development (**Figure 3B**).

Furthermore, we investigated the spatiotemporal expression pattern of *SEMA5A* in the mouse embryo at embryonic day 14.5, on images of ISH from GenePaint. Consistent with the findings mentioned above, it showed that *SEMA5A* was strongly expressed in the neocortex of the embryonic mouse brain (**Figure 3C**). The expression profiles of *SEMA5A* in the brain indicates that this gene may play an essential role in the development of the brain and that dysfunctional *SEMA5A* may lead to human neurodevelopmental disorders such as EEs.

### PPI Network and Gene Function Analyses

**Figure 4:**
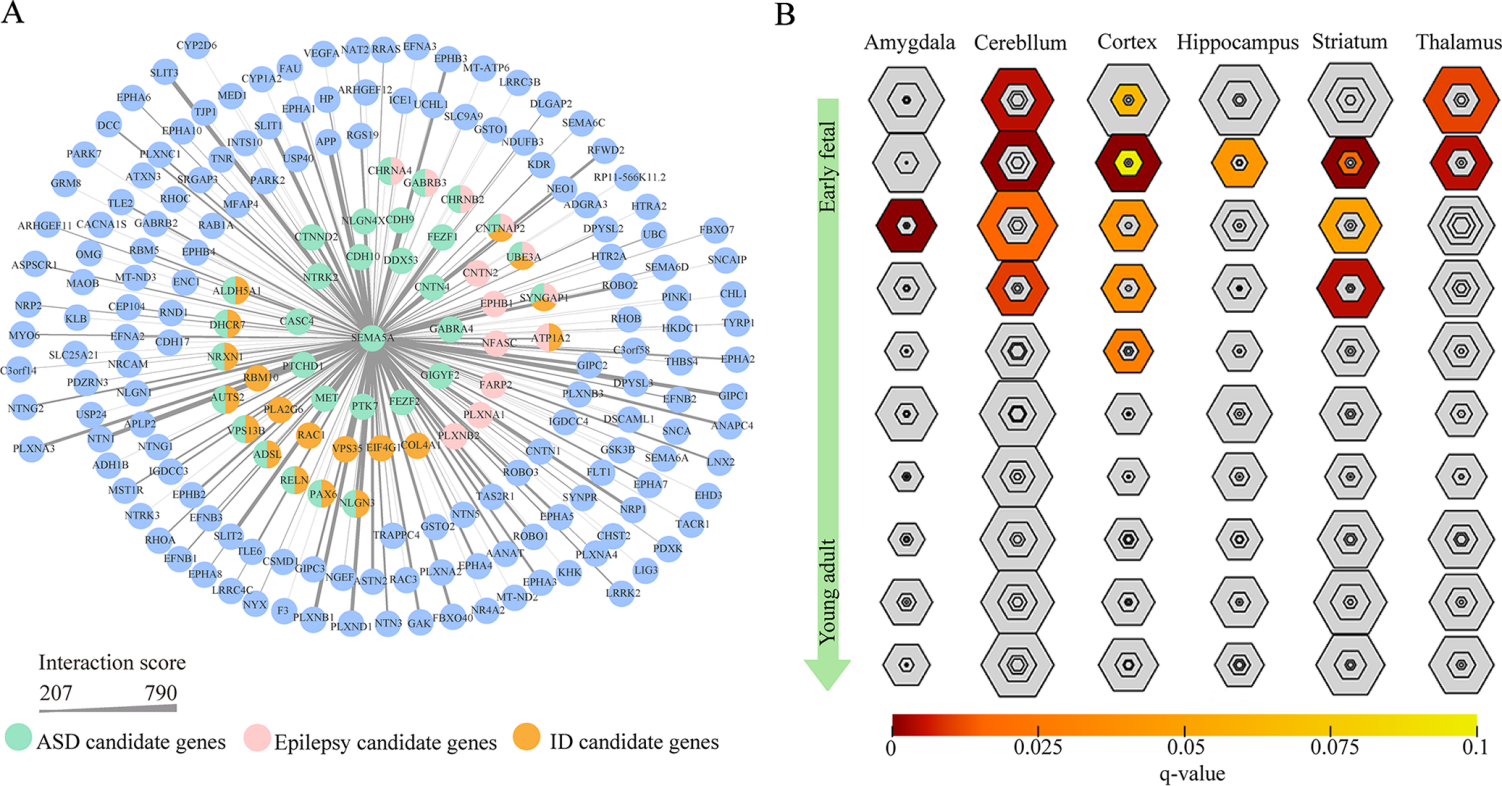
Protein–protein interaction (PPI) network and specific expression analysis (SEA) enrichment analysis. **(A)** PPI network of genes found to interact with *SEMA5A* in the STRING database and to have interaction scores greater than 200. The nodes represent genes, and edges represent the intersection between gene pairs. The thickness of an edge denotes the interaction scores between gene pairs. **(B)** SEA of genes in the PPI network. Each hexagon shows different stringency thresholds, with colors indicating q values.

To gain insight into the biological function of *SEMA5A* and those genes that interact with *SEMA5A*, we extracted genes with interaction scores greater than 200 from the STRING database, and then constructed a PPI network. In the resulting PPI network, we found 196 nodes, among which 43 genes were known candidate genes in neurodevelopmental disorders. These 43 genes included 16 ASD candidate genes, six epilepsy candidate genes, six intellectual disability (ID) candidate genes, three candidate genes shared by ASD and epilepsy, nine shared by ASD and ID, one shared by ID and epilepsy and three shared by ASD, ID and epilepsy (**Figure 4A**). To evaluate the interconnectivity among these 43 genes, we performed an interconnected PPI analysis and observed a non-random interaction connectivity with 100,000 permutation tests (q = 1 × 10^−5^ for genes, q = 1 × 10^−5^ for connections). Moreover, we discovered that these genes were significantly enriched in PSD (q = 7.50 × 10^−6^) and genes under evolutionary constraint (q = 2.04 × 10^−5^) (**Supplementary Figure S3 and S4**).

**Figure 5:**
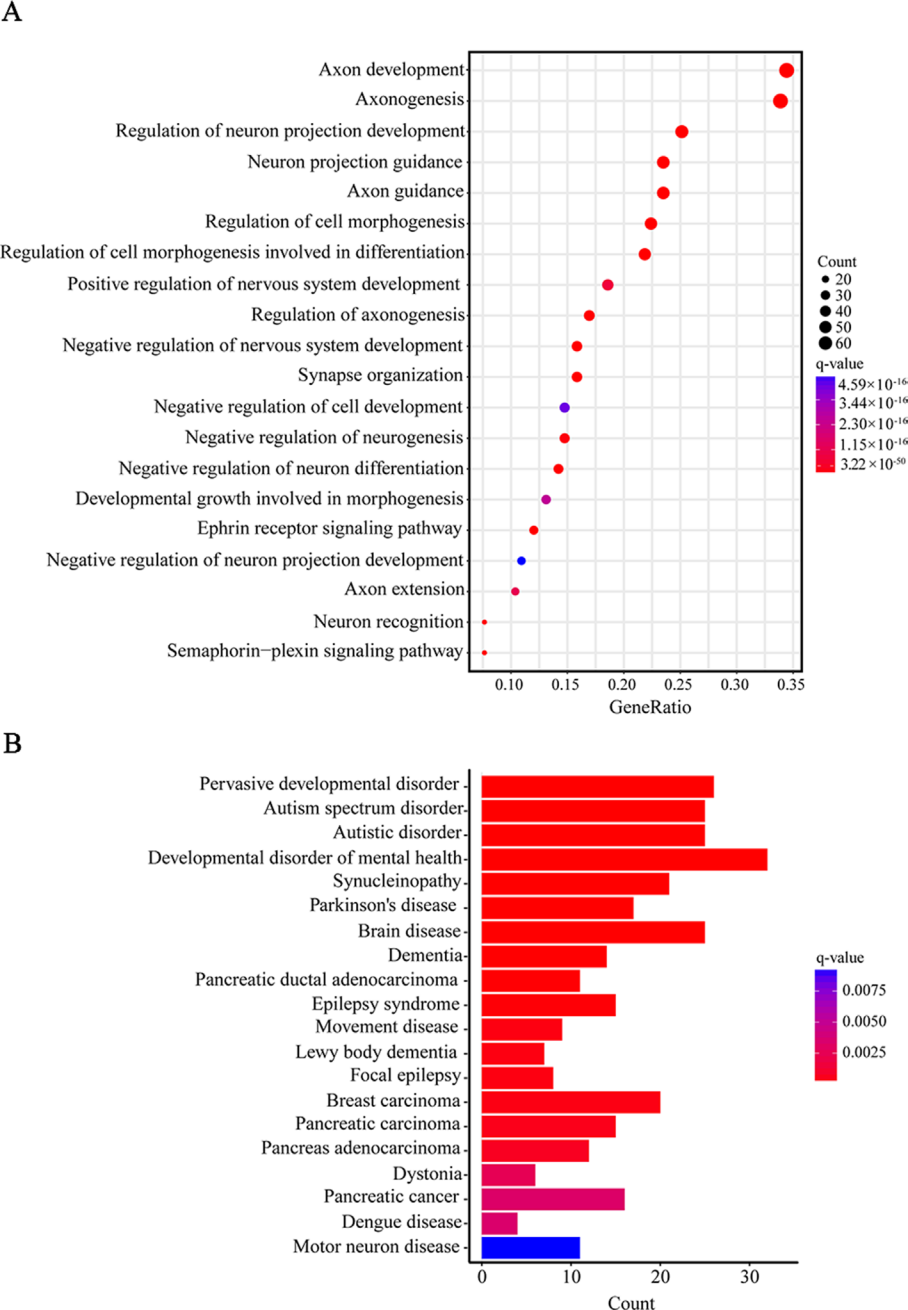
Gene Ontology (GO) and Disease Ontology (DO) enrichment analyses. **(A)** The top 20 enriched GO terms of biological process. Circle size indicates the number of genes enriched in each term. Color saturation represents the significance level. **(B)** The top 20 terms in the DO enrichment analysis. The x-axis shows the number of genes enriched in each term. The q-value of each term is indicated by color according to the legend.

Furthermore, when we performed a specific spatiotemporal expression analysis to further explore the expression patterns of the interacting genes, as expected, we observed that *SEMA5A* and its interacting genes were significantly enriched in the cortex, cerebellum, striatum and thalamus from early fetal to early infancy periods (q < 0.05) (**Figure 4B**). Next, a Gene Ontology (GO) enrichment analysis was performed to determine whether the genes in the PPI network were enriched in neurodevelopment-associated GO terms. The results showed that *SEMA5A* and its interacting genes were significantly enriched in axon-, neuron- and synapse-associated GO terms, such as axon development (q = 2.05 × 10^−49^), axon guidance (q = 5.22 × 10^−39^), the regulation of neuron projection development (q = 4.86 × 10^−31^), and synapse organization (q = 2.73 × 10^−19^) (**Figure 5A**). Moreover, it is important to note that genes in the PPI network were relevant to ASD (q = 1.88 × 10^−13^), developmental disorders of mental health (q = 2.48 × 10^−12^), Parkinson’s disease (q = 1.61 × 10^−6^) and epilepsy syndrome (q = 8.92 × 10^−5^) based on Disease Ontology (DO) analysis (**Figure 5B**).

## Discussion

In this study, we performed WES on three trios and presented evidence showing that a *de novo* missense mutation in *SEMA5A* was likely associated with IS. Considering the potential role of recessive inheritance in EEs, we also tried to detect homozygous inherited SNVs or indel mutations in other genes in our samples. However, there were no candidate damaging compound mutations or homozygous mutations in all patients except a compound heterozygous mutation in *CLTCL1* in patient 2. *SEMA5A* belongs to the semaphorin gene family, the members of which serve as canonical axon guidance proteins and function in pathological conditions of the nervous system (Yaron and Zheng, 2007). It is now clear that semaphorins and their receptors play many crucial roles in the development of neural circuits (Pasterkamp, 2012), including roles in neuronal migration (Hernandez-Miranda et al., 2011), axon bundling (Van Battum et al., 2015), axon pruning (Pasterkamp, 2012) and synaptic transmission (Sahay et al., 2005; Carrillo et al., 2010). Moreover, some alterations in the expression of axon guidance proteins, such as semaphorins and ephrins, have been observed in animal models of epilepsy (Barnes et al., 2003; Xia et al., 2013). It is generally accepted that semaphorins that can act as axon guidance proteins are involved in a variety of neurological diseases, including ASD (McFadden and Minshew, 2013), epilepsy (Xia et al., 2013), Parkinson’s disease (Lin et al., 2009) and Alzheimer’s disease (Good et al., 2004).

Moreover, semaphorins are sensitive to electrical activity and experience and form a large family consisting of eight classes, among which class 5 semaphorins (*SEMA5A* and *SEMA5B*) play essential roles in the functions of the developing nervous system (Mann et al., 2007). SEMA5A, as an integral membrane protein, acts as a bifunctional guidance cue that could be directly implicated in both attractive and inhibitory processes during axon development (Kantor et al., 2004). Furthermore, *Sema5A* was shown to be essential for the proper development of fasciculus retroflexus in a rat model (Kantor et al., 2004). A separate study showed that *Sema5A* functions as a negative regulator of synaptogenesis during early brain development and synaptic transmission (Duan et al., 2014).

Cumulative evidence obtained from various sources shows that *SEMA5A* is a transmembrane protein that has been identified as an autism susceptibility gene in humans according to a genome-wide association study (Weiss et al., 2009), cDNA microarray technology (Melin et al., 2006) and expression quantitative trait locus mapping (Cheng et al., 2013). Notably, a *de novo* microdeletion in *SEMA5A* was identified in a patient with ASD and ID (Mosca-Boidron et al., 2016). It is worth noting that ASD and EEs often occur together and share some common genetic etiologies (Srivastava and Sahin, 2017). Abnormal synaptic plasticity may represent a common pathophysiological mechanism in ASD and epilepsy that can lead to an imbalance between excitatory and inhibitory neurotransmission in the developing brain (Brooks-Kayal, 2010). *NRP2* is a receptor for both *SEMA3C* and *SEMA3F*, each of which plays an important role in axon guidance in the peripheral and central nervous systems (Chen et al., 2000) in addition to synaptic plasticity (Lee et al., 2012). Positional and functional evidence has been indicated that polymorphisms and mutations in *NRP2* may be associated with autism (Wu et al., 2007). For example, *NRP2* knockout mice were found to have a lowered seizure threshold and were more sensitive to chemical challenges aimed at inducing epileptogenesis (Gant et al., 2009). Notably, several studies have demonstrated that *SEMA3C* and *SEMA3F*, which are homologous to *SEMA5A*, are candidate genes for EEs (Barnes et al., 2003; Mefford et al., 2011). The patient carrying damaging DNM in *SEMA5A* in this study was diagnosed with IS at the age of only 7 months, though there was no behaviors or phenotypes related to ASD based on clinical diagnosis, which may be due to the very young age of the patient. Therefore, it is necessary to continue examinations or follow-up surveys for this patient as they become older, to evaluate whether ASD develops.

*CLTCL1*, a clathrin heavy chain protein in humans, is associated with the neuromuscular system (Towler et al., 2004), and neuropeptide degradation and secretion during neuronal development (Nahorski et al., 2018). The homozygous R125C mutation in *CLTCL1*, inherited from heterozygous parents, was predicted to be damaging and has been identified in patients with autism (Chahrour et al., 2012). A mutation in *CLTCL1* located on chromosome 22q11.2 has also been associated with susceptibility to schizophrenia (Karayiorgou et al., 2010; Chahrour et al., 2012). DiGeorge syndrome is usually associated with the deletion of chromosome 22q11.2, which is linked with cognitive impairments, susceptibility to schizophrenia and neuroanatomical changes (Karayiorgou et al., 2010). It is worth noting that an interruption in the *CLTCL* gene observed in a patient with DiGeorge syndrome was found to contribute to the patient’s phenotype, including a seizure disorder, ID and facial dysmorphia (Holmes et al., 1997). However, whether the compound mutation in *CLTCL* identified in this study was associated with IS still required further genetic evidence or functional studies to support it.

## Conclusions

To our knowledge, this is a novel DNM of *SEMA5A* found in an individual with IS. Our results provide genetic and functional evidence showing that *SEMA5A* plays a significant role in the development of the brain and might be involved in several neurodevelopmental disorders, such as EE and ASD. The discovery of damaging mutations in *SEMA5A* provides further evidence supporting the role of axon guidance proteins in the pathogenesis and causes underlying EE. However, to definitively establish the role of *SEMA5A* in IS, further genetic function studies are needed.

## Ethics Statement

This study was carried out with the approval provided by the Second Affiliated Hospital and Yuying Children’s Hospital of Wenzhou Medical University. Written informed consent of all participants was collected from their parents or guardians at the time of recruitment for participation in the study and publication of the study.

## Author Contributions

QW and ZWL contributed to the drafting and revision of the manuscript, data acquisition, and analysis. ZDL, RZ, YL, and FL contributed to individual recruitment, data acquisition, and data analysis. XX, MT, and WS contributed to data acquisition and manuscript revision. XZ, JZ, and YL contributed to study concept and design, critical review, and manuscript revision.

## Funding

This study was supported by the Zhejiang Provincial Natural Science Foundation of China (Grant No. LY18C060007).

## Conflict of Interest Statement

The authors declare that the research was conducted in the absence of any commercial or financial relationships that could be construed as a potential conflict of interest.
