# Supplementary material for: De Novo Germline Mutations in SEMA5A Associated With Infantile Spasms
Source: Front Genet. 2019 Jul 10;10:605. doi: 10.3389/fgene.2019.00605 (PMC6635550; doi:10.3389/fgene.2019.00605)
Supplement: Supplementary file 1 [file Table_1.docx]

**Table S1. Statistics for WES**

| **Sample** | **Clean data**  **(Gb)** | **Aligned (%)** | **Base covered on target**  **(Mb)** | **Fraction of effective bases on target (%)** | **Average sequencing depth on target** | **4X**  **coverage**  **(%)** | **10X coverage**  **(%)** | **20X coverage**  **(%)** |
| --- | --- | --- | --- | --- | --- | --- | --- | --- |
| Patient 1P | 6.62 | 99.56 | 46.91 | 61.70 | 77.03 | 96.40 | 93.30 | 86.40 |
| Patient 1F | 5.90 | 99.55 | 46.91 | 58.40 | 64.78 | 96.40 | 93.50 | 85.50 |
| Patient 1M | 5.97 | 99.43 | 46.85 | 62.80 | 71.10 | 96.00 | 91.90 | 83.50 |
| Patient 2P | 7.00 | 99.20 | 46.84 | 55.10 | 67.71 | 96.20 | 93.10 | 85.70 |
| Patient 2F | 11.03 | 99.33 | 46.66 | 57.90 | 109.95 | 95.60 | 92.50 | 87.40 |
| Patient 2M | 9.49 | 99.39 | 46.71 | 55.40 | 88.57 | 95.90 | 92.90 | 87.10 |
| Patient 3P | 5.58 | 99.58 | 47.53 | 54.60 | 58.78 | 98.40 | 96.50 | 89.40 |
| Patient 3F | 7.86 | 99.51 | 47.59 | 54.50 | 83.42 | 98.70 | 97.70 | 94.30 |
| Patient 3M | 5.22 | 99.08 | 47.41 | 54.00 | 52.89 | 98.10 | 96.00 | 88.10 |
| Mean | 7.19 | 99.40 | 47.05 | 57.16 | 74.91 | 96.86 | 94.16 | 87.49 |
