# Supplementary figures and images for: De Novo Germline Mutations in SEMA5A Associated With Infantile Spasms
Source: Front Genet. 2019 Jul 10;10:605. doi: 10.3389/fgene.2019.00605 (PMC6635550; doi:10.3389/fgene.2019.00605)

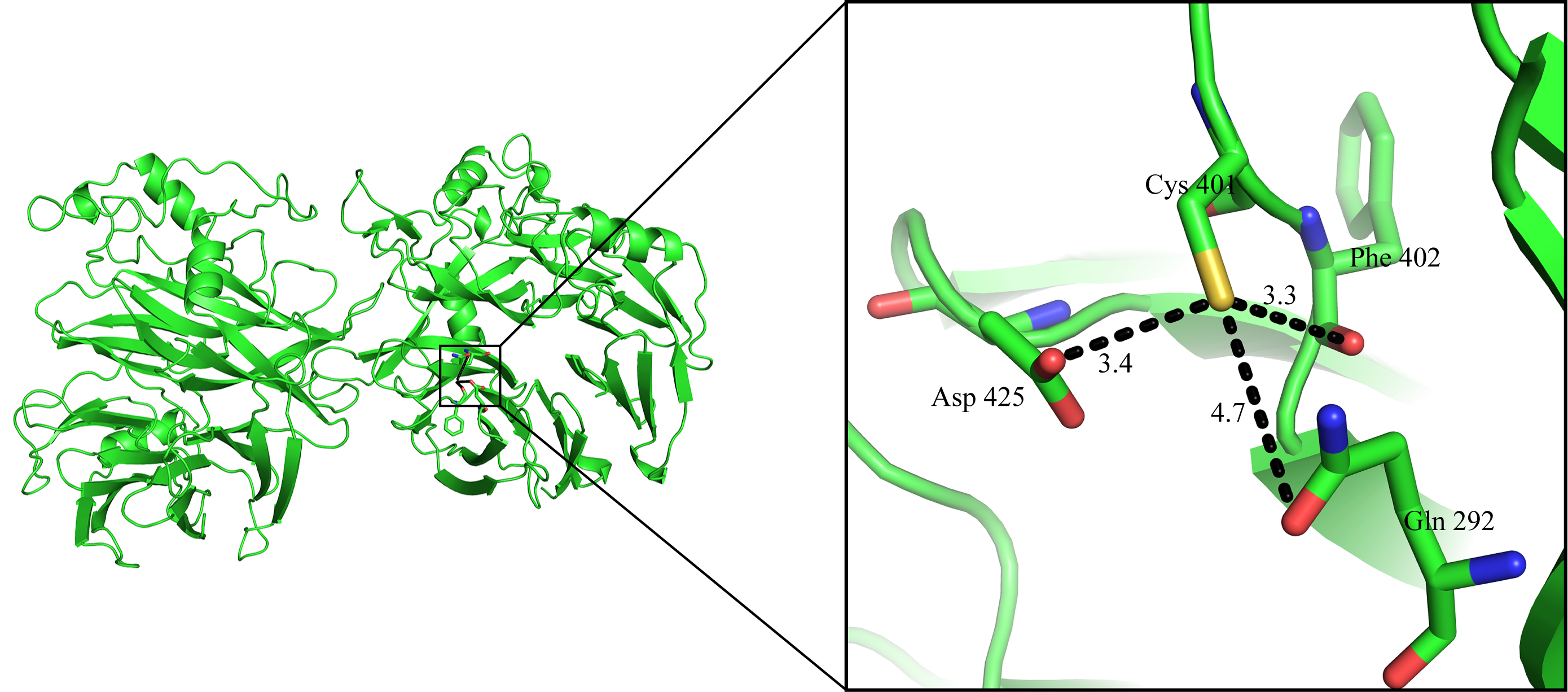

Supplement: Supplementary file 3 [file Image_1.tif]

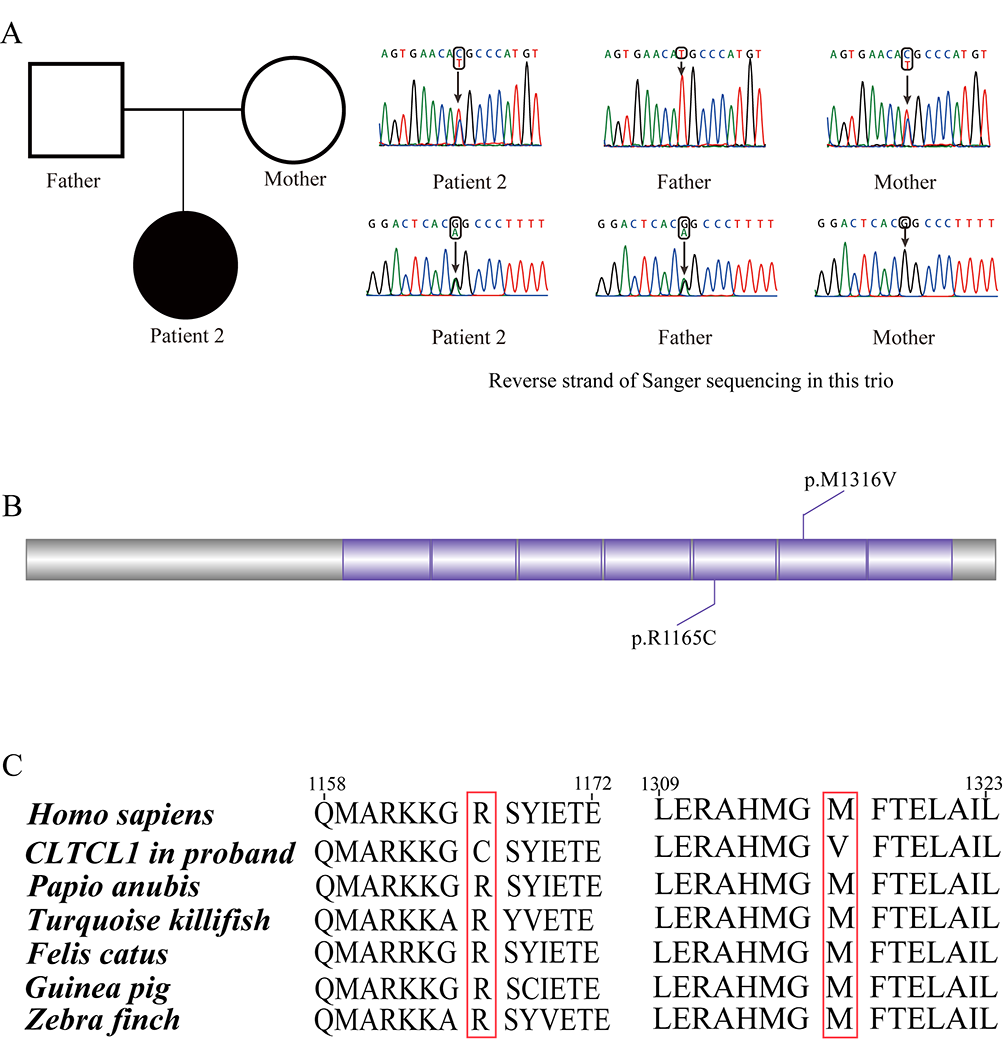

Supplement: Supplementary file 4 [file Image_2.tif]

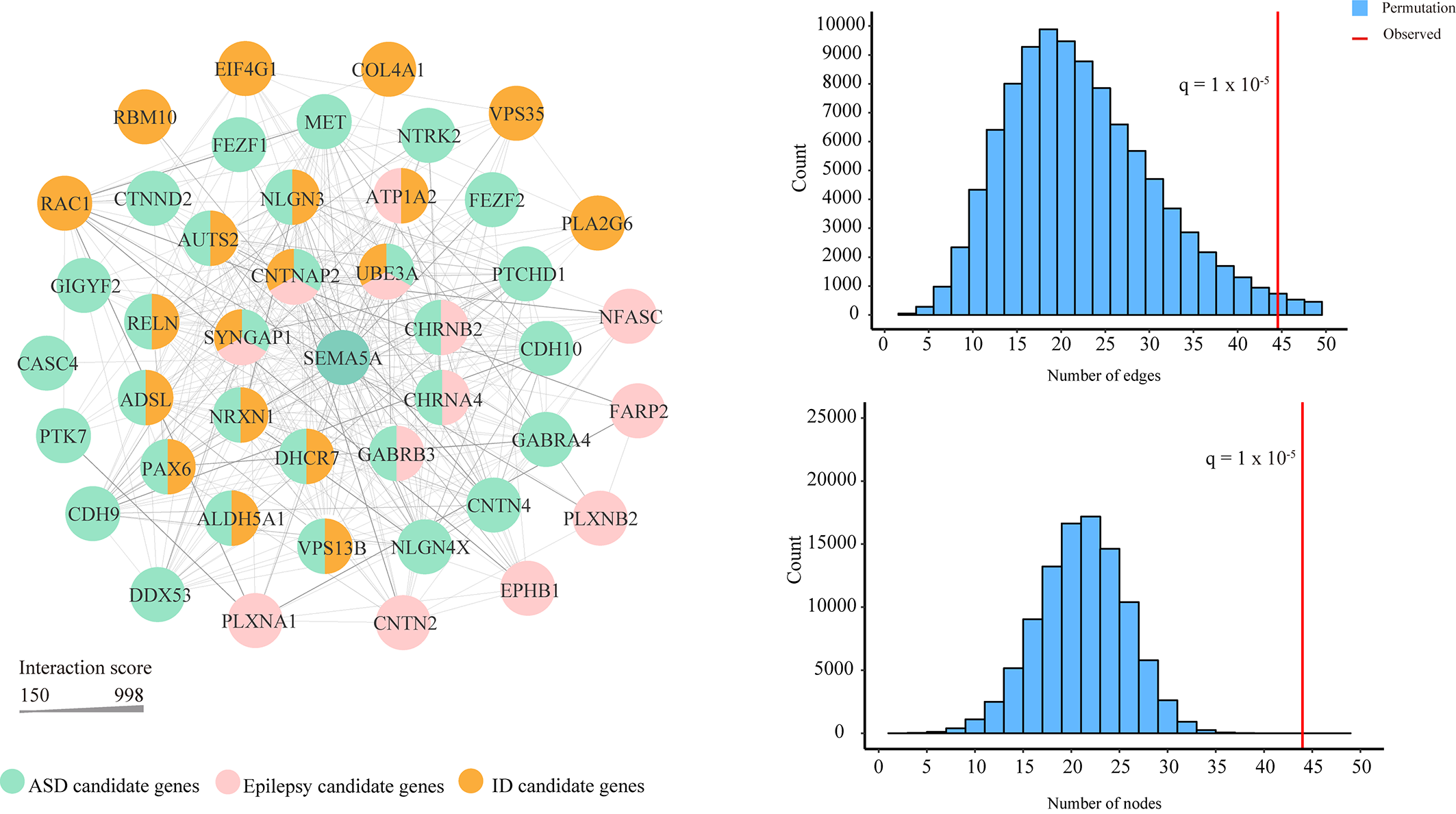

Supplement: Supplementary file 5 [file Image_3.tif]

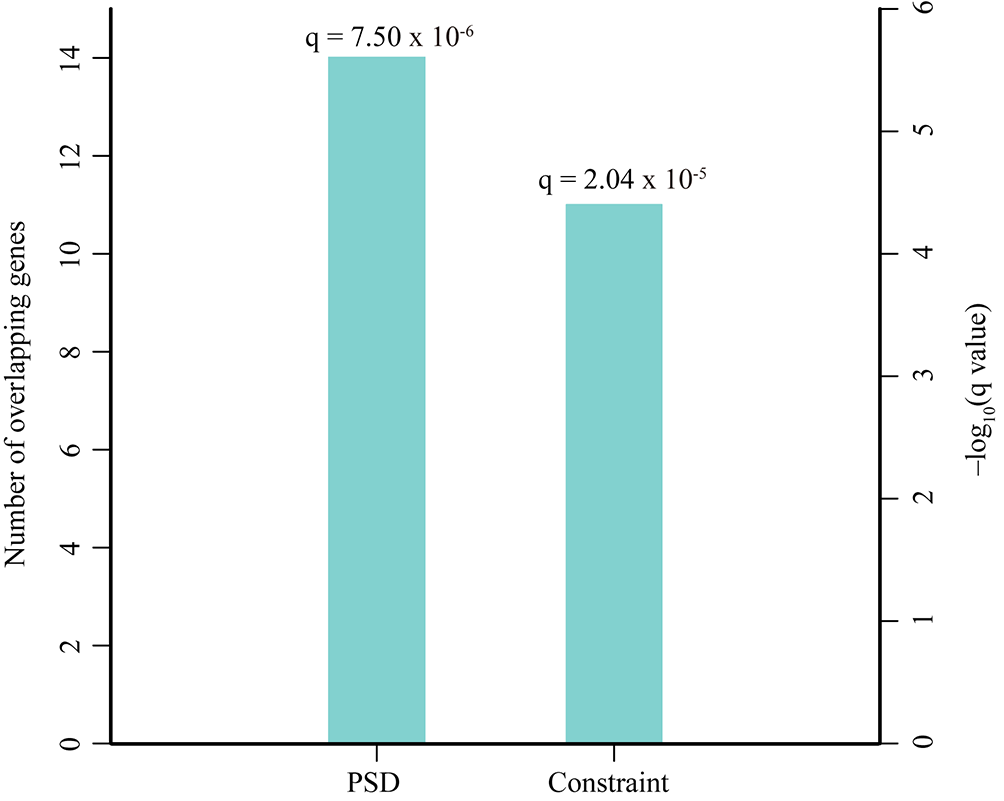

Supplement: Supplementary file 6 [file Image_4.tif]
